# The clinical significance and underlying correlation of pStat-3 and integrin αvβ6 expression in gallbladder cancer

**DOI:** 10.18632/oncotarget.14444

**Published:** 2017-01-02

**Authors:** Liu Enyu, Wang Na, Zhao Chuanzong, Wang Ben, Wu Xiaojuan, Wang Yan, Li Zequn, Hong Jianguo, Wang Jiayong, Liang Benjia, Peng Cheng, Zhu Min, Zhang Zongli

**Affiliations:** ^1^ Department of General Surgery, Qilu Hospital of Shandong University, Jinan 250012, Shandong, P.R. China; ^2^ Department of Dermatology, Shandong Provinical Institute of dermatology and venereology, Jinan 250012, Shandong, P.R. China; ^3^ Department of Pathology, School of Medicine, Shandong University, Jinan 250012, Shandong, P.R. China; ^4^ Key Laboratory of Cardiovascular Remodeling and Function Research, Chinese Ministry of Education and Public Health, Jinan 250012, Shandong, P.R. China

**Keywords:** gallbladder cancer, integrin β6, pStat-3, immunohistochemistry, tumorigenesis

## Abstract

**Background:**

Both phosphorylated signal transducer and activator of transcription 3(pStat-3) and integrin αvβ6 can play vital role in the development and progression of cancer. However, little is known about their expression correlation and clinical significance in gallbladder cancer(GBC).

**Objective:**

The aim of our present study was to investigate the expression of pStat-3 and integrin αvβ6, two proteins’ correlation and their clinical significance in GBC tissues.

**Results:**

The expression of pStat-3 and integrin αvβ6 were both significantly associated with T stage, lymph node metastasis status, TNM stage (P=0.008, P=0.000, P=0.000 and P=0.036, P=0.001,P=0.000,respectively). IHC and Western blot showed their expressions in GBC tissues were higher than that in paraneoplastic tissues. Moderate positive correlation existed between the two proteins (r =0.349, P <0.001). The survival analysis by Kaplan-Meier and Cox regression model showed that GBC patients with pStat-3 or integrin αvβ6 positive expression had a significantly poorer 2-year survival rate (P = 0.002 and 0.000, the log-rank test, respectively), and either marker could act as unfavorable independent prognostic factors(RR=1.907, P=0.021 and RR=2.046, P=0.038).

**Materials and Methods:**

The expression levels of pStat-3 and integrin αvβ6 were analyzed in GBC cancerous and paraneoplastic tissues of 97 cases via immunohistochemistry(IHC) and further validated by western blot method. Besides, SPSS software was used to observe their clinical significance as well as the two proteins’ correlation.

**Conclusion:**

pStat-3 and integrin αvβ6 were indicators of tumor's progression and poor prognosis of patients with GBC. And the further study involving them may provide a helpful therapeutic target in prevention and treatment of GBC patients.

## INTRODUCTION

Gallbladder cancer (GBC) is a highly aggressive malignant tumor of the biliary tract, which ranks fifth common among gastrointestinal cancers and accounts for 46% of biliary tract cancers in the United States [1]. Meanwhile the incidence rate of GBC is now increasing worldwide, with a rather poor prognosis [2]. However, most of these patients are diagnosed at an advanced stage lacking chances of radical surgery [3], and only about 10% were detected at early stage [4]. Therefore, it is important to explore underlying molecular mechanism of GBC for improve prognosis of GBC patients.

Generally known, long-term chronic inflammation predisposes tissue to cancer development in the process of GBC tumorigenesis [5–7]. Current studies have shown that signal transducer and activator of transcription 3 (STAT3) is a definitive link between inflammation and cancer transformation [8]. As a transcriptional factor, phosphorylation of STAT3 can subsequently promote transcriptions of target genes involving inflammation and malignancy. Consequently, persistent Stat3 target gene activation can stimulate cell growth, angiogenesis, metastasis, and anti-apoptosis, thereby driving and sustaining tumorigenesis [9–11].

Recent evidence has proved that STAT3 is involved in promoting transcription of integrin beta6 in oral squamous cell carcinoma and prostate epithelial cell caicinoma cells [12, 13]. As is known, integrin families(24 members compromising of 18 α-subunits and 8 β-subunit) can mediate cellular adhesion to extracellular matrix (ECM) and modulate diverse process such as cell proliferation, migration, invasion, and survival by activating intracellular signaling pathways [14–16]. Integrin β6, with its sole binding partner αv subunit, is an epithelial-cell-restricted antigen, which is expressed in tissue remodeling events (e.g., fibrosis, wound healing), as well as epithelial cancers (e.g., lung, breast, pancreas, ovary, oropharynx and colon) [17]. Integn αvβ6 plays an vital role in various aspects of cancer progression and tissue repairing [18, 19].

However, the expression relationship of STAT3 and integrin αvβ6 in gallbladder cancer, and their underlying interaction at molecular level have not been investigated before. The aim of our present study was to explore the expression and clinical significance of STAT3 combined with integrin αvβ6 in GBC, which could provide a novel strategy for further study of molecular mechanism in therapeutic intervention.

## RESULTS

### The expression of pStat-3 and integrin αvβ6 in patients with gallbladder adenocarcinoma

**Figure 1:**
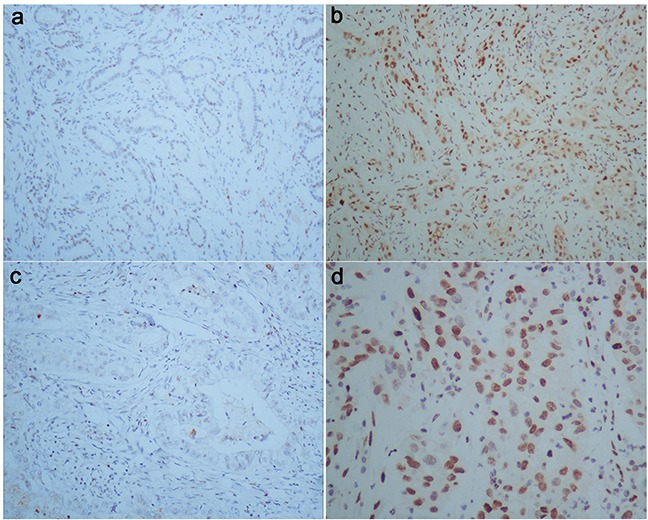
pStat-3 expression in human gallbladder carcinoma First row: **a, b.** (Magnification 100 X);second row: **c, d.** corresponding to each figure above (Magnification 200 X, 400 X, respectively). (a) and (c) were obtained from paracancerous normal tissue of patient with GBC, with negative staining. (b) and (d) were obtained from GBC tissue, with positive pStat-3 expression.

**Figure 2:**
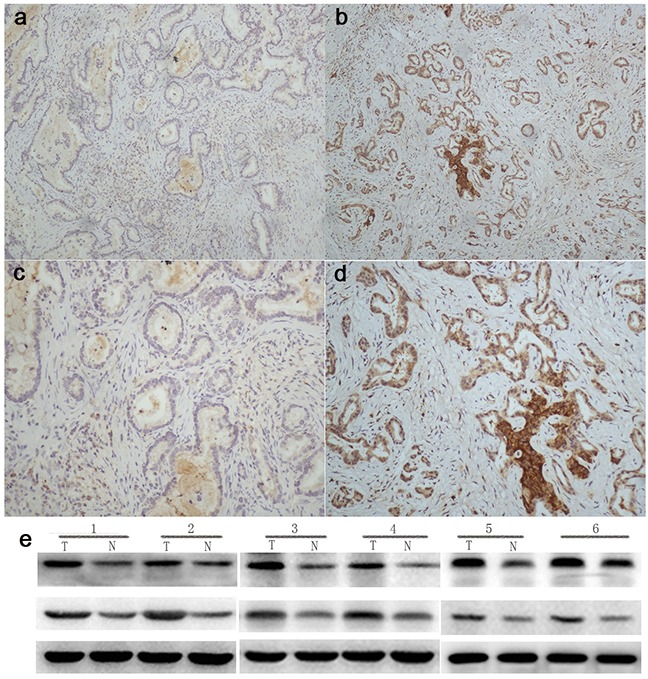
Integrinβ6 expression in human gallbladder carcinoma First row: **a, b**. (Magnification 100 X);second row: **c, d**. (Magnification 200 X). (a) and (c). were obtained from paracancerous normal tissue of patient with GBC, with negative staining. (b) and (d). were obtained from GBC tissue, with negative integrinβ6 expression. **e**. To further validate the expression of αvβ6 and pStat-3 in GBC, we performed western blot analysis, which indicated the expression of both proteins in tumor tissues were significantly higher than that in corresponding paracancerous normal tissue.

Immunohistochemistry results showed that pStat-3 was detected mainly in the nuclei, as well as in the cytoplasm. And integrinαvβ6 was expressed both in cytoplasm and on cellular membrane. For pStat-3 expression, 59 of 97 cases with GBC could express pStat-3 in tumor tissue, with positive rate of 60.8%, while 38 ones could not be stained as negative expression (Figure 1, Table 1). For integrin αvβ6 patients, 44 of 97 cases(45.4%) were positive expression, and 53 were negative (Figure 2, Table 1). And in the normal peritumoral tissues for the two markers were all negative staining(P<0.001). Moreover, we could find only 5 pStat-3 positive expression and 2 integrinαvβ6 positive expression exsiting in chronic cholecystitis tissues of 36 patients, significantly lower than that in tumor tissues.

**Table 1:** Association between pStat-3 expression, Integrinαvβ6 expression and clinicopathologic variables in gallbladder cancer patients

Clinicopathological factors	n	pStat-3 expression		P Value	Integrin αvβ6 expression		P Value
		Positive (n=59)	Negative (n=38)		Positive (n=44)	Negative (n=53)	
Gender				0.565			0.271
Male	34	22	12		18	16	
Female	63	37	26		26	37	
Age(years)				0.577			0.426
<60	35	20	15		14	21	
>=60	62	39	23		30	32	
T stage				0.008 ^#^			0.036 ^#^
T1	9	3	6		4	5	
T2	43	23	20		13	30	
T3	35	23	12		20	15	
T4	10	10	0		7	3	
Lymph node metastasis				0.000			0.001
yes	36	30	6		30	6	
no	61	29	32		14	47	
TNM stage				0.000 ^#^			0.000 ^#^
I	6	1	5		1	5	
II	26	7	19		1	25	
III	48	35	13		30	18	
IV	17	16	1		12	5	
Differentiation				0.778			0.268
well	46	26	20		17	29	
moderate	37	24	13		19	18	
Poor/undifferentiated	14	9	5		8	6	
Gallstone				0.147			0.423
No	55	30	25		23	32	
Yes	42	29	13		21	21	
Pathological type				0.117			0.336
adenocarcinoma	79	45	34		34	45	
Squamous/ adenosquamous carcinoma	18	14	4		10	8	
Survival (24-month follow-up)				0.002*			0.000*
death	79	52	27		37	42	
censored	18	7	11		7	11	

* Log-rank test; ^#^ Fisher's Exact Test

Furthermore, we performed western blot analysis to validate the expression of αvβ6 and pStat-3 in GBC tissue, we performed western blot analysis in 10 paired primary tumor tissue and corresponding paracancerous nontumorous tissue, which indicated the expression of both proteins in 6 tumor tissues were significantly higher than that in their corresponding normal tissues among all the 10 GBC patients, as shown in Figure 2.

### Correlations of pStat-3 and integrinαvβ6 expression with clinicopathologic features of gallbladder adenocarcinoma

pStat-3 positive staining is significantly higher in advanced T stage and TNM stages(P=0.008, P=0.000, respectively). Meanwhile, lymph node metastasis also affected pStat-3 staining, the pStat-3 positive rate of specimens with lymph node metastasis was 83.3%, much higher than that of no lymph node metastasis group 47.5%(P=0.000). As for integrin αvβ6, significant association also existed between positive integrinαvβ6 expression and advanced T stage (P= 0.036),TNM stage (P= 0.000), as well as lymph node metastasis status (P= 0.001). However, there was no significant association among pStat-3 expression, patients’ gender and age, pathological grade and type, and gallstone status, which was similar in patients’ integrinαvβ6 expression. The detailed clinicopathologic data for pStat-3 and integrinαvβ6 are outlined in Table 1.

### pStat-3 or integrinαvβ6 expression and the survival of patients with GBC

**Figure 3:**
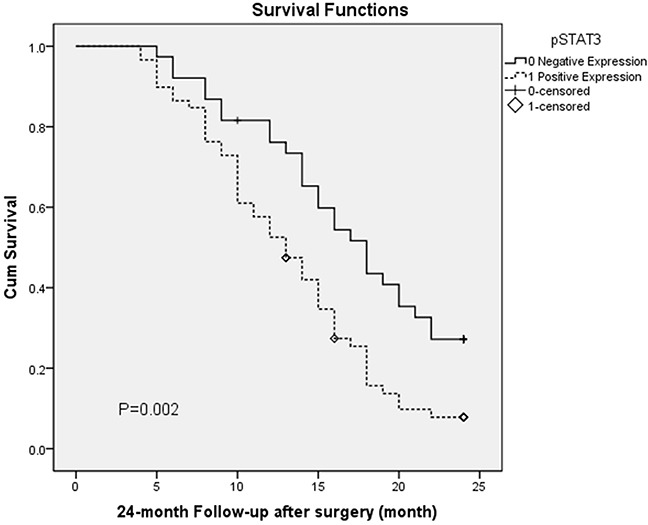
Overall survival according to pStat-3 expression (P = 0.002, The log-rank test)

**Figure 4:**
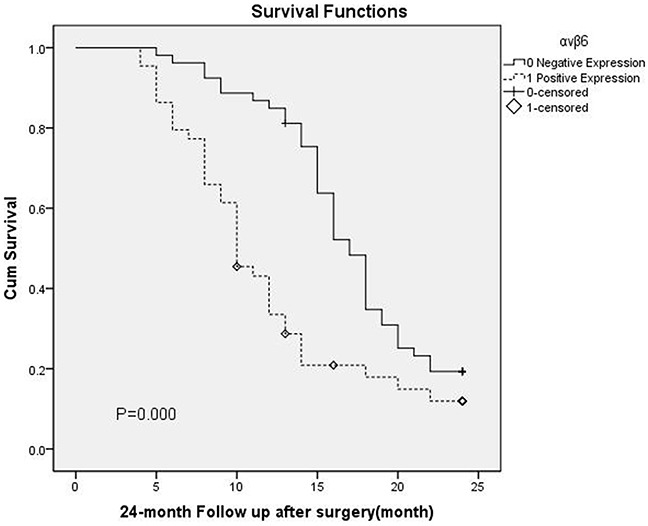
Overall survival according to αvβ6 expression(P = 0.000, The log-rank test)

All the 97 patients with gallbladder carcinoma were followed up for 24 month follow-up was conducted after surgery through email or phones. Kaplan-Meier survival analysis showed that patients with positive pStat-3 or integrinαvβ6 expression had a significantly poorer overall survival than those with negative expression (P = 0.002, P=0.000, The log-rank test, respectively). Patients’ survival over time on pStat-3 or integrinαvβ6 expression is illustrated in Figure 3 and Figure 4.

### The correlation between integrinαvβ6 and pStat-3 expression in patients with GBC

As shown in Table 2, 35 of 44 patients(79.5%) with positive integrin αvβ6 expression was observed in positive pStat-3 expression tissue, while 29 of 53 patients(54.7%) with negative integrin αvβ6 expression existed in negative pStat-3 expression tissue. According to the Spearman correlation analysis, integrinαvβ6 expression had a moderate positive correlation with pStat-3 expression in patients with GBC(r =0.349,P <0.001, Table 2).

**Table 2:** Correlation between integrinαvβ6 expression and pStat-3 expression in human gallbladder carcinoma tissues (Spearman correlation, r = 0.349, P < 0.001)

pStat-3	integrin αvβ6		total
	Positive	Negative	
Positive	35	24	59
Negative	9	29	38
total	44	53	97

**Figure 5:**
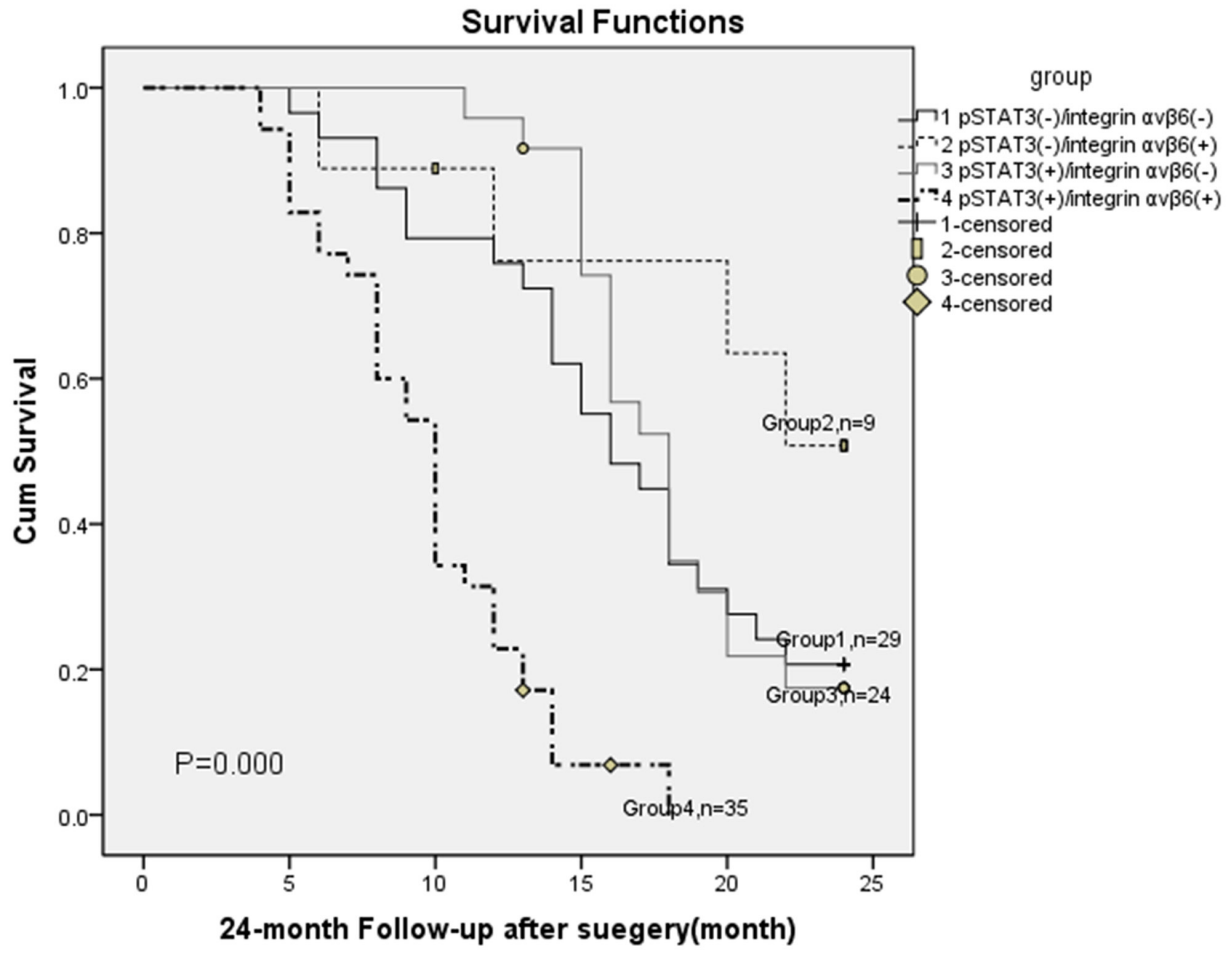
Overall survival according to pStat-3 with integrin αvβ6 expression (P = 0.028, The log-rank test)

These 97 cases with GBC were classified into 4 groups according to integrinαvβ6 and pStat-3 expression, as follows: Group 1 with pStat-3(−) / integrinαvβ6(−) (n = 29); Group 2 with pStat-3(−) / integrinαvβ6(+) (n =9); Group 3 with pStat-3(+) / integrinαvβ6(−) ( n =24 ); and Group 4, pStat-3(+)/integrinαvβ6(+) ( n = 35). Patients with pStat-3(+)/integrinαvβ6(+) had a significantly poorer overall survival rate than other groups (P = 0.000, The log-rank test), which is illustrated in Figure 5.

### Univariate and multivariate analysis for prognosis of patients with gallbladder cancer

Cox proportional hazards regression model was applied to perform univariate and multivariate analyses to determine the prognostic value of clinicopathologic factors, pStat-3 and integrinαvβ6 expression. In univariate analysis, positive expression of pStat-3 andαvβ6, plus advanced T stage, lymph node metastasis status and TNM stage could predict a poorer prognosis (P=0.004, P=0.001, P=0.028, P=0.002, P=0.011,respectively)(Shown in Table 3). Next, these variables with P<0.05 were collected for multivariate analysis, results revealed that positive pStat-3 andαvβ6 expression were unfavorable independent prognostic factors for patients with GBC( relative risk (RR): 1.907 and 2.046;P=0.021 and 0.038, respectively) (Shown in Table 3).

**Table 3:** Univariate and multivariate analysis of association of clinicopathologic features with survival of GBC patients

Variable	Univariate Analysis			Multivariate Analysis		
	Relative Risk	95% CI	P Value	Relative Risk	95% CI	P Value
Age at Diagnosis	0.978	0.950, 1.007	0.137			
gender						
Male	1.000 (Ref.)	-	-			
Female	1.311	0.810, 2.122	0.270			
T stage			0.028			0.487
T1	1.000 (Ref.)	-	-	1.000 (Ref.)	-	-
T2	2.673	0.948,7.532	0.063	1.610	0.447,5.797	0.466
T3	4.287	1.502,12.236	0.007	2.500	0.685,9.126	0.166
T4	3.652	1.092,12.217	0.036	2.297	0.424,12.456	0.335
Lymph node metastasis						
no	1.000 (Ref.)	-	-	1.000 (Ref.)	-	-
yes	2.027	1.291,3.183	0.002	1.359	0.630,2.934	0.434
TNM stage			0.011			0.521
I	1.000 (Ref.)	-	-			0.238
II	6.780	0.911,50.470	0.062	4.067	0.396,41.773	0.368
III	13.027	1.783,95.153	0.011	3.039	0.270,34.238	0.536
IV	10.208	1.340,77.791	0.025	2.250	0.172,29.374	
Pathology Grade			0.166			
Well	1.000 (Ref.)	-	-			
Moderate	1.581	0.976,2.559	0.062			
Poor/undifferentiated	1.406	0.711,2.782	0.328			
Gallstone						
No	1.000 (Ref.)	-	-			
Yes	1.134	0.726,1.771	0.582			
Pathological type						
adenocarcinoma	1.000 (Ref.)	-	-			
Squamous/ adenosquamous carcinoma	1.044	0.602,1.808	0.879			
pStat-3						
negative expression	1.000 (Ref.)	-	-	1.000 (Ref.)	-	-
positive expression	1.991	1.240,3.197	0.004	1.907	1.103,3.297	0.021
αvβ6						
Negative	1.000 (Ref.)	-	-	1.000 (Ref.)	-	-
Positive	2.174	1.383,3.419	0.001	2.046	1.041,4.022	0.038

## DISCUSSION

Gallbladder cancer is a very aggressive malignant tumor with rising incidence and poor clinical outcome [20]. Despite improvements made in diagnosis and treatments during the past years, the prognosis of GBC remains extremely unsatisfactory [2, 5]. Therefore, understanding and exploring the molecular mechanism of tumorigenesis is in urgency, which would contribute to treatment of patients with this disease. In our present study, the positive expression of pSTAT-3 and integrinαvβ6 were observed via IHC staining and Western blot method in GBC samples. And we found that both p-STAT3 and integrinαvβ6 expression were significantly correlated with clinicopathological features and survival time for patients with GBC.

As is reported, integrinαvβ6 could be expressed in many epithelial cancers, including lung, breast, pancreas, ovary, oropharynx and colon cancers, as well as during tissue remodeling events such as fibrosis and wound healing [21, 22]. However, the expression of integrin αvβ6 in GBC has never been investigated before. In this study, for the first time, we found that integrinαvβ6 could be expressed in GBC cancerous tissues, and the expression rate was 45.4%. Also, our results showed that its expression were significantly associated with clinical TNM stage, lymph node metastasis and the depth of invasion of tumors. According to the Cox regression model and survival analysis, we could prove that integrinαvβ6 predicted a poor prognosis for GBC patients, the same as shown in patients with colonic and gastric carcinomas. We have previously shown a direct linkage between ERK2 and the cytoplasmic domain of β6, through which the increasing phosphorylation of ERK2 could effectively contribute to the activation of downstream targets, involved in regulation of cellular invasion, metastasis, anti-apoptosis, chemo resistance and degradation of extracellular matrix by mediating MMP-9 secretion [23–26].

In present study, we first found p-Stat3 was obviously expressed in cancerous tissue of GBC, and its expression was also significantly associated with clinical TNM stage, T stage and lymph node metastasis. And the Cox regression model and survival analysis showed that pStat could also predict a poor prognosis for GBC patients. As a transcriptional factor, STAT3 can be activated through phosphorylation at tyrosine 705 (Y705) and serine 727 (S727) by inflammatory cytokines driven signaling including interleukin-6(IL-6), IL-10, IL-17, IL-21, IL-23, and vascular endothelial growth factor [27]. And phosphorylation of STAT3 can subsequently promote transcriptions of target genes involving inflammation and malignancy, such as Bcl-2, Bcl-xL, Mcl-1, Fas, cyclin D1, c-myc, VEGF, HIF-1, TGFβ, and TIMP-1,etc [28]. Normally, Stat3 is activated in a transient manner, while it can be constitutively activated in long-term inflammatory and cancerous cells [8]. As a result, persistent Stat3 target gene activation can promote tumor development and sustain tumorigenesis [29].

Importantly, in this study, we found moderate positive correlation exists between p-Stat3 and integrin αvβ6 by Spearman correlation analysis. To some extent, it demonstrated that certain interaction and relationship exist between protein expression levels of the two genes. This result is just in line with recent evidence that ITGB6 promoter contains a functional TATA box located −289 to−150 and that is binding sites for transcription factors STAT3 in oral squamous cell carcinoma cells [12]. Namely, integrin beta6 was downstream target genes of STAT3, which was involved in positive regulation of integrin beta6 transcription. In fact, integrin beta6 expression could be primarily regulated at the level of transcription initiation [30, 31], as well as at translational level [32]. It has been reported that under certain conditions, transcription factors including Ets-1 [30], STAT3 [12, 13], Smad3, AP-1 [33]and C/EBPα [12]could promote the initiation of integrin beta6 expression.

We have known that the long-term chronic inflammation of gallbladder is a high risk factor for tumorigenesis of GBC, which could predispose inflammatory tissue to cancer development [20, 34]. The continuous evidence of recent years has convinced us that IL-6/JAK/STAT3 pathway can play a definitive role in the transformation from inflammation to cancer for colorectum tumor [35]. Phosphorylation of Stat3 at Y705 could be facilitated by JAK family, and it is required for Stat3 homo- or hetero-dimerization, nuclear translocation, and DNA binding [17]. Moreover, cytokine-driven JAK/STAT3 pathways are involved in cellular proliferation, differentiation, invasion, survival, and even inflammation and immune function of various human cancers [8, 11]. Taken together, we could make a reasonable hypothesis that cytokine-driven JAK/STAT3 pathways may also mediate the expression of integrin beta6, which play an important role in tumor development and progression of GBC. Of course, our further work aims to demonstrate our hypotheses in cell lines and animal model. Promisingly, targeting the IL-6/JAK/STAT3 pathway may provide potential strategies for prevention and treatment of GBC.

## MATERIALS AND METHODS

### Patients and follow-up

We collected data and tumor specimens from patients diagnosed with gallbladder carcinoma(GBC) at Qilu Hospital of Shandong University between July 2006 and January 2013. All petients with GBC included in this study have received surgical resection(radical or palliative type) as the initial treatment modality without major perioperative complications, and also had enough archived tissue kept at Pathological Department of hospital. This resulted in a collection of tumor tissue from 97 patients, 34 males and 63 females with a median age of 61.3 years and an age range of 40–73 years. And for comparison, we also collected 76 benign gallbladder tissue samples including 40 peritumoral normal tissues taken from 97 GBC patients mentioned above, and the other 36 chronic cholecystitis tissues. These malignant and benign gallbladder carcinoma specimens were all formalin-fixed and paraffin-embedded. The pathologic tumor–node–metastasis(TNM) classification was based on the criteria of the 7th edition American Joint Committee on Cancer(AJCC), and the tumor stage was confirmed by expert pathologists.

All the 97 patients with gallbladder carcinoma were followed up for 24 months after surgery through email or phones. Among those, 79(81.4%) were confirmed GBC-associated death within 24 months of prognosis, 14 patients were alive beyond 60 months, and 4 were censored as their case follow up was discontinued or died of reasons other than GBC. The mean survival time for these patients was 14.4 months.

All patients or relatives signed an informed consent of this study. It was approved by the Ethics Committee of Qilu Hospital, Shandong University. The details of patients’ characteristics have been listed in Table 1.

### Antibodies and immunohistochemistry

Immunohistochemistry was performed to evaluate the expression of pStat-3 and αvβ6 on 4 μm thickness Paraffin-embedded tissue sections. The rabbit-anti-human monoclonal antibody EP2147Y (1:150,Abcam,Cambridge UK)against STAT3 (phospho Y705) and goat-anti-human polyclonal C-19 agaist integrin β6 from Santa Cruz (1:200,Santa Cruz, USA) were applied respectively. HRP secondary antibodies and DAB kit were obtained from Zhongshan Golden Bridge (Beijing, China).

Then, immunohistochemical staining for the two markers was done as follows. The sections were deparaffinized and hydrated, and the heat induced antigen epitope retrieval was performed using microwave method, then and the slides were immersed in EDTA antigen retrieval solution (pH 9.0) for 20 min. Subsequently, 3% hydrogen peroxide was added to inhibit endogenous peroxidase activity. Next, antibodies STAT3 (1:150; Abcam,Cambridge, UK), integrin β6 (1:200,Santa Cruz, USA) were applied to the sections, which were later incubated by either antibody at 4°C overnight. On the second day, tissue sections were incubated with universal IgG antibody-Fab-HRP polymer (Zhongshan Biotech, Beijing China)for 30 min; next, DAB and Hematoxylin were used to stain, and after sealed with neutral tree gum sequentially, sections were prepared for visualization under light microscope (Olympus Corp, Tokyo, Japan).

### Evaluation of pStat-3 and integrin αvβ6 immunohistochemistry

It is known that integrinαvβ6 was expressed both in cytoplasm and on cellular membrane, mainly seen on the internal surface of the tumor epithelial cell membrane. And pStat-3 can Shuttle between the nucleus and the cytoplasm, which predominantly presents in the cytoplasm without stimuli, and accumulates in the nucleus upon stimulation. We evaluated the expression levels of pStat-3 and integrin αvβ6 via semi-quantitative method based on the average intensity and percentage of positively stained tumor cells. The score of intensity was graded as follows: 0 (no staining), 1 (weak staining, light yellow), 2 (moderate staining, yellow brown), and 3 (strong staining, brown). And the percentage of stained tumor cells was scored as (0, 0%; 1, <20%; 2, 20~50%; 3, 51~75%; 4, > 75%). Then we defined the staining class according to the consensus results of both scores as follows: < 2, negative expression; 2~4, low (weak) expression, and ≥5, high (strong) expression. And both low and high expressions were graded as αvβ6 and pStat-3 positive.

### Western blot analysis

To further analyze and validate the expression of αvβ6 and pStat-3 in GBC, western blot analysis was performed in 10 paired fresh primary tumor tissue and corresponding paracancerous normal tissue. And these paired fresh specimen were collected immediately after surgery between January 2011 and November 2012 and were stored at −80°C. All patients have confirmed the informed consent.

For Western Blot Analysis, total proteins extracted from fresh tissues were prepared in radio immunoprecipitation assay (RIPA) buffer (Beyotime, Jiangsu, China) including complete protease inhibitor cocktail (Roche Applied Science, Mannheim, Germany). Total proteins were separated by 10% SDSPAGE and then transferred to PVDF membranes. The membranes were blocked with 5% skim milk in Trisbuffered saline with 0.1% Tween-20 (TBST) for 1 h at room temperature and then incubated with anti- pStat-3 (1:1000; Abcam, Cambridge, UK), anti-integrinβ6 (1:1500, Santa Cruz, USA) or anti-GAPDH (1:1000; Abcam, Cambridge, UK) antibodies overnight at 4°C. After incubation with horseradish peroxidase conjugated anti-rabbit secondary antibodies for 1 h, proteins were detected using enhanced chemiluminescence (Millipore, Billerica, MA).

### Statistical analysis

Statistical analyses were performed with SPSS 13.0 software. The relationship among expression of pStat-3, integrinαvβ6 and their clinicopathologic characteristics was examined using χ^2^ test or Fisher exact test. Survival analyses were carried out via the Kaplan-Meier method. And the Cox proportional hazard model was used to conduct univariate and Multivariate analysis. Besides, the relationship between pStat-3 and integrin αvβ6 expression levels was evaluated by Spearman correlation. *P* < 0.05 was considered statistically significant.

## CONCLUSION

Our findings indicated that the expression levels of pSTAT3 and αvβ6 are up-regulated in GBC tissue, which were associated with tumor progression and poor prognosis of patients. Besides, moderate-poor correlation existed between the expression of pSTAT3 and integrin αvβ6, and molecular mechanism underlying them may contribute to the transformation from inflammation to cancer of GBC, which may provide a potential therapeutic approach to treat this disease.
